# Prognostic value of serial score measurements of the national early warning score, the quick sequential organ failure assessment and the systemic inflammatory response syndrome to predict clinical outcome in early sepsis

**DOI:** 10.1097/MEJ.0000000000000924

**Published:** 2022-06-23

**Authors:** Lara E.E.C. Zonneveld, Raymond J. van Wijk, Tycho J. Olgers, Hjalmar R. Bouma, Jan C. ter Maaten

**Affiliations:** Departments ofa Internal Medicine; b Clinical Pharmacy and Pharmacology, University Medical Center Groningen, University of Groningen, Groningen, The Netherlands

**Keywords:** clinical deterioration, emergency department, national early warning score, quick sequential organ failure assessment, repeated measurements, sepsis, systemic inflammatory response syndrome

## Abstract

Sepsis is a common and potentially lethal syndrome, and early recognition is critical to prevent deterioration. Yet, currently available scores to facilitate recognition of sepsis lack prognostic accuracy.

**Objective:**

To identify the optimal time-point to determine NEWS, qSOFA and SIRS for the prediction of clinical deterioration in early sepsis and to determine whether the change in these scores over time improves their prognostic accuracy.

**Design:**

Post hoc analysis of prospectively collected data.

**Settings and participants:**

This study was performed in the emergency department (ED) of a tertiary-care teaching hospital. Adult medical patients with (potential) sepsis were included.

**Outcome measures and analysis:**

The primary outcome was clinical deterioration within 72 h after admission, defined as organ failure development, the composite outcome of ICU-admission and death. Secondary outcomes were the composite of ICU-admission/death and a rise in SOFA at least 2. Scores were calculated at the ED with 30-min intervals. ROC analyses were constructed to compare the prognostic accuracy of the scores.

**Results:**

In total, 1750 patients were included, of which 360 (20.6%) deteriorated and 79 (4.5%) went to the ICU or died within 72 h. The NEWS at triage (AUC, 0.62; 95% CI, 0.59–0.65) had a higher accuracy than qSOFA (AUC, 0.60; 95% CI, 0.56–0.63) and SIRS (AUC, 0.59; 95% CI, 0.56–0.63) for predicting deterioration. The AUC of the NEWS at 1 h (0.65; 95% CI, 0.63–0.69) and 150 min after triage (0.64; 95% CI, 0.61–0.68) was higher than the AUC of the NEWS at triage. The qSOFA had the highest AUC at 90 min after triage (0.62; 95% CI, 0.58–0.65), whereas the SIRS had the highest AUC at 60 min after triage (0.60; 95% CI, 0.56–0.63); both are not significantly different from triage. The NEWS had a better accuracy to predict ICU-admission/death <72 h compared with qSOFA (AUC difference, 0.092) and SIRS (AUC difference, 0.137). No differences were found for the prediction of a rise in SOFA at least 2 within 72 h between the scores. Patients with the largest improvement in any of the scores were more prone to deteriorate.

**Conclusion:**

NEWS had a higher prognostic accuracy to predict deterioration compared with SIRS and qSOFA; the highest accuracy was reached at 1 h after triage.

## Introduction

Sepsis is a life-threatening, dysregulated host response to infection leading to organ dysfunction and potentially death [1,2]. Globally, 48.9 million sepsis cases and 11 million sepsis-related deaths were reported in 2017, representing 19.7% of all global deaths [3]. Approximately 21% of all adults visit the emergency department (ED) because of a serious infection, of which 11% classify as severe sepsis with an inhospital mortality of 13% [4]. Current treatment consists of fluid resuscitation, antibiotics and, if needed, vasoconstrictive medication, supplemental oxygen and organ support. Delay in the administration of antibiotics to patients with severe sepsis is associated with an increased risk of organ damage [5,6] and mortality [7]. Antibiotic treatment within 4 h in patients with nonsevere sepsis is associated with a reduced length of stay and mortality [8]. Additionally, early fluid resuscitation and administration of norepinephrine to patients with severe sepsis lower the mortality rate [9,10]. Early recognition that is essential to initiate adequate treatment on time is critical to avert organ damage and death.

Different clinical scoring systems are available to facilitate early sepsis recognition: most used are the quick Sequential Organ Failure Assessment (qSOFA), the systemic inflammatory response syndrome (SIRS)-criteria and the national early warning score (NEWS). The SIRS has a relatively good sensitivity (86%) to predict mortality at the cost of a relatively low specificity (29%). In contrast, the qSOFA has a better specificity (83%) but a poor sensitivity (51%) to predict inhospital mortality [11,12]. The NEWS balances between the SIRS and qSOFA: the sensitivity for mortality or ICU admission is 74% sensitivity, and the specificity for these outcomes is 43% [13]. Their clinical applicability in early sepsis, however, is limited due to the low prognostic accuracy to predict ICU-admission and mortality, as well as the lack of insight in the prognostic accuracy to predict other clinically relevant outcomes such as progression of organ dysfunction. In order to adequately support clinical decision making at the ED, it is essential to be aware of the prognostic accuracy of sepsis scores to predict progression of organ dysfunction and need of escalated care. Currently, it is unknown at which moment the sepsis scores should be calculated, even though the patients' clinical condition is dynamic over time. Evidence about which time-point is best in predicting deterioration and the importance of a trend in scores is scarce [14].

Thus, sepsis is a common and potentially lethal syndrome, and early recognition is critical to allow timely initiation of adequate treatment to prevent organ failure. Yet, available scores to facilitate recognition of early sepsis lack discriminative value and their accuracy at different moments in time to predict (progressive) organ dysfunction are not known. The aim of this study was to identify the optimal time-point to calculate NEWS, qSOFA and SIRS with regard to predicting deterioration, defined as organ failure, ICU admission and mortality, and to assess whether their change over time improves prognostic value for deterioration by analyzing serial measurements at the ED.

## Methods

### Study design and setting

This study is a post hoc analysis, based on a prospective observational study (Sepsis Clinical Pathway Database) at the ED of the University Medical Center Groningen (UMCG), a tertiary-care teaching hospital [15]. As ruled by the Institutional Review Board of the UMCG, the Dutch Medical Research Involving Human Subject Act is not applicable for this study, and a waiver was granted (METc 2015/164). All participants provided written informed consent.

### Study population

Data were collected between March 2016 and July 2020. Medical patients visiting the ED between 08.00 and 21.00 h were screened for inclusion. The inclusion criteria entailed: (a) being 18 years or older, (b) fever (≥38 °C) or suspected infection or sepsis (judged by the coordinating internist acute medicine), and (c) being able to provide informed consent. Only patients with at least one vital sign measurement, besides triage, were included. All patients received treatment according to protocol.

### Data collection

All data were collected by trained research staff at the ED during the patients' stay, to avoid bias due to interobserver variability. Afterward, the database was complemented using electronic medical records. Vital parameters were measured every half hour. Based on these, qSOFA, NEWS and SIRS were calculated during the first 4 h (or until ED discharge).

### Missing data

Missing values were estimated by calculating the average from the measurement directly 30 min before and directly 30 min after the missing value. If either one was absent, data were not calculated. Missing half-hour values of the Glasgow Coma Scale-scores and supplemental oxygen were deemed normal or not present if missing after calculating the average. The remaining missing values were calculated by using the multiple imputations function of SPSS.

### Endpoint definitions

The primary outcome was deterioration within 72 h after ED admission. Deterioration was defined as the development of (multi)organ failure; distinguished as acute kidney injury (AKI), liver failure and/or respiratory failure, ICU-admission or mortality. AKI was defined using the kidney disease improvement global outcomes criteria as an increase in serum creatinine 1.5 times the baseline (presumed or occurred within the last 7 days) and/or an increase of 26.5 ≥ µmol/l within 48 h [16]. The occurrence of SOFA score of at least 1 within the same category compared with the score at the ED was used to define liver failure (bilirubin > 32 µmol/l) and respiratory failure (PaO_2_/FiO_2_ ≤ 300), and the partial pressure of oxygen divided by the fractional inspired oxygen (P/F-ratio), without respiratory support or PaO_2_/FiO_2_ ≤ 200 with respiratory support [17]. Our secondary outcome was ICU-admission/mortality less than 72 h. For comparison with the standard method, a rise in SOFA score of at least two points was added as the third outcome.

### Statistical analysis

Continuous data were reported as median with interquartile range and analyzed using the Mann–Whitney *U* test. Categorical data were summarized as counts with percentages and analyzed using the Chi-square test. The relationship between scoring systems and deterioration was assessed by using the area under the receiver operating characteristics (AUROC) curve. The areas under the ROC (AUCs) were compared using the ‘paired-sampled area difference under the ROC curve’ function of SPSS. Post hoc power analysis via a univariate model was used to determine the power of the used sample size. Sensitivities, specificities, and positive and negative predictive values were calculated. Post hoc power analysis was used to evaluate the sample size. Statistical analyses were performed using IBM SPSS Statistics version 26 (Armonk, New York, USA).

## Results

### Patient characteristics

**Fig. 1:**
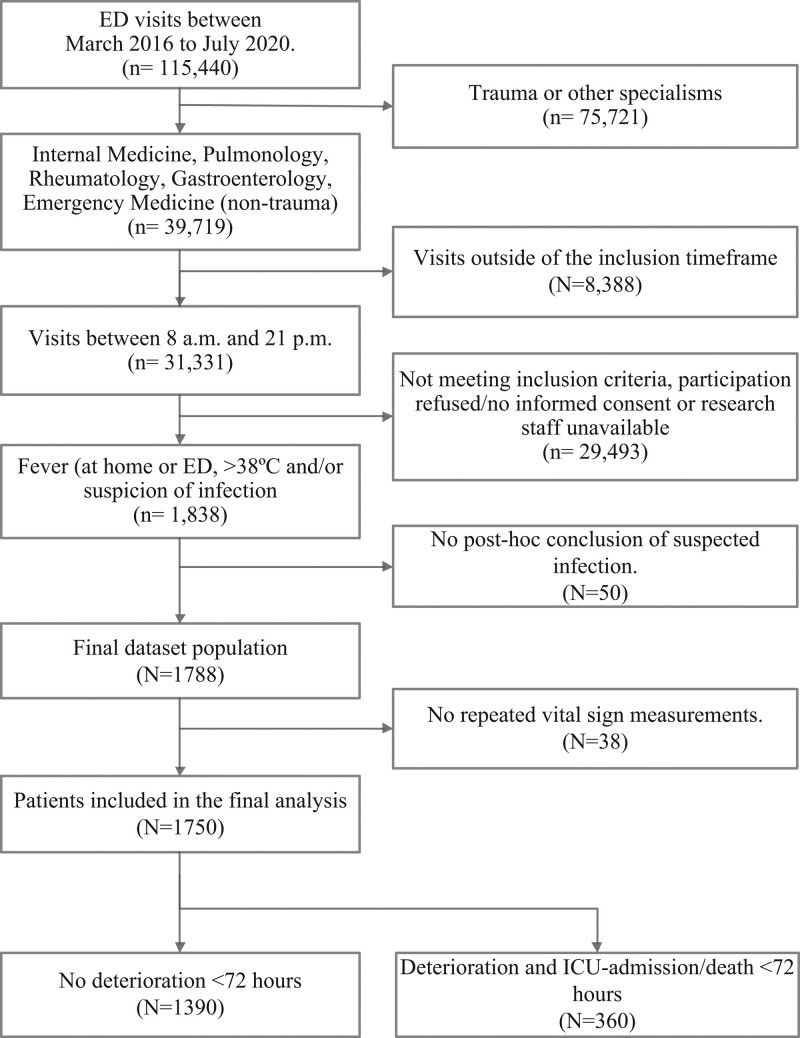
Flowchart of patient selection. Adult medical patients visiting the emergency department (ED) between March 2016 and July 2020 were screened for inclusion. Deterioration: death, ICU-admission, development of new kidney failure (defined following the kidney disease improvement global outcomes criteria), respiratory failure [an increase of one of more points in the sequential organ failure assessment (SOFA) score for this category] and liver failure (an increase of one of more points in the SOFA score for this category) within 72 h after admission to the ED.

In total, 1750 patients were included, of which 360 (20.6%) deteriorated within 72 h, 68 (3.9%) necessitated ICU-admission and 15 (0.9%) died within 72 h (Fig. 1 and Table 1). Patients who deteriorated were on average older (*P* < 0.001) and were more often tobacco (*P* = 0.003) or alcohol users (*P* = 0.029), and a higher number of patients had chronic obstructive pulmonary disease (*P* = 0.002), diabetes (*P* = 0.009) or chronic kidney disease (*P* = 0.009). The length of stay in hospital was longer among patients who deteriorated (median, 7 and 3 days, respectively, *P* < 0.001) (Table 1).

**Table 1:** Main characteristics of the study population *N* = 1750

Variable (% missing)	Overall	No deterioration <72 h	Deterioration <72 h	*P*-value	No ICU or mortality <72 h	ICU or mortality <72 h	*P*-value
Number of patients (*n*)	1750	1390 (79.4)	360 (20.6)	ND	1671 (95.5)	79 (4.5)	ND
Demographics							
Age (0.0) [median (IQR)]	63 (53–73)	62 (51–63)	67 (58.1–75.9)	<0.001*	63 (52.5–73.5)	68 (59–77)	0.001*
Male (0.0) [*n* (%)]	997 (57.0)	781 (44.6)	216 (12.3)	0.193	949 (54.2	48 (2.7)	0.486
Living at home (4.0) [*n* (%)]	1533 (87.6)	109 (6.5)	38 (2.3)	0.083	133 (7.9)	14 (0.8)	0.001*
Smoker^1^ (7.1) [*n* (%)]	240 (13.7)	174 (10.7)	66 (4.1)	0.003*	227 (14.0)	13 (0.8)	0.330
Alcohol user^2^ (7.7) [*n* (%)]	469 (26.8)	389 (24.1)	80 (5.0)	0.029*	454 (28.1)	15 (0.9)	0.151
Comorbidity [*n* (%)]							
Cardiac disease (1.9)	315 (18.0)	240 (14.0)	75 (4.4)	0.123	298 (17.4)	17 (1.0)	0.388
COPD (0.0)	159 (9.1)	111 (6.3)	48 (2.7)	0.002*	151 (8.6)	8 (0.5)	0.674
Diabetes (1.7)	409 (23.4)	306 (17.8)	103 (6.0)	0.009*	384 (22.3)	25 (1.5)	0.078
Chronic kidney disease (1.9)	251 (14.3)	184 (10.7)	67 (3.9)	0.009*	237 (13.8)	14 (0.8)	0.366
Chronic liver disease (0.0)	237 (13.5)	198 (11.3)	39 (2.2)	0.092	234 (13.4)	3 (0.2)	0.010*
Organ transplant (2.1)	300 (17.1)	238 (13.9)	62 (3.6)	0.956	293 (17.1)	7 (0.4)	0.047*
Malignancy (1.1)	698 (39.9)	552 (31.9)	146 (8.4)	0.767	660 (38.1)	38 (2.2)	0.099
None of the above (1.8)	368 (21.0)	308 (17.9)	60 (3.5)	0.022*	355 (20.7)	13 (0.8)	0.322
Scoring systems^3^ [median (IQR)]							
SOFA (0.0)	2 (1–3)	1 (0–2)	2 (0.5–3.5)	<0.001*	1 (−1 to 1)	4 (0–8)	<0.001*
qSOFA (0.0)	0 (−0.5 to 0.5)	0 (−0.5 to 0.5)	1 (0.5–1.5)	<0.001*	0 (−0.5 to 0.5)	1 (−0.5 to 0.5)	<0.001*
NEWS (0.0)	3 (1–5)	2 (0–4)	4 (2–6)	<0.001*	3 (1–5)	7 (2–12)	<0.001*
SIRS (0.0)	2 (0–4)	2 (1–3)	2 (0–4)	<0.001*	2 (0–4)	3 (2–4)	<0.001*
Length of stay (days) (0.0) [median (IQR)]	4 (2–6)	3 (−0.5 to 6.5)	7 (3–11)	<0.001*	4 (0–8)	8 (2.5–13.5)	<0.001*

Deterioration; death, ICU-admission or development of organ failure (respiratory, liver and/or kidney) <72h. 1≥1 cigarette a day; 2≥1 unit of alcohol per week; 3 during triage; Percentages of total is shown within parentheses.

COPD, chronic obstructive pulmonary disease; IQR, interquartile range; NEWS, national early warning score; ND, no data; qSOFA, quick sequential organ failure assessment; SIRS, systemic inflammatory response syndrome.

* *P* < 0.05.

### Sepsis scoring systems per time-point as predictor of deterioration

**Fig. 2:**
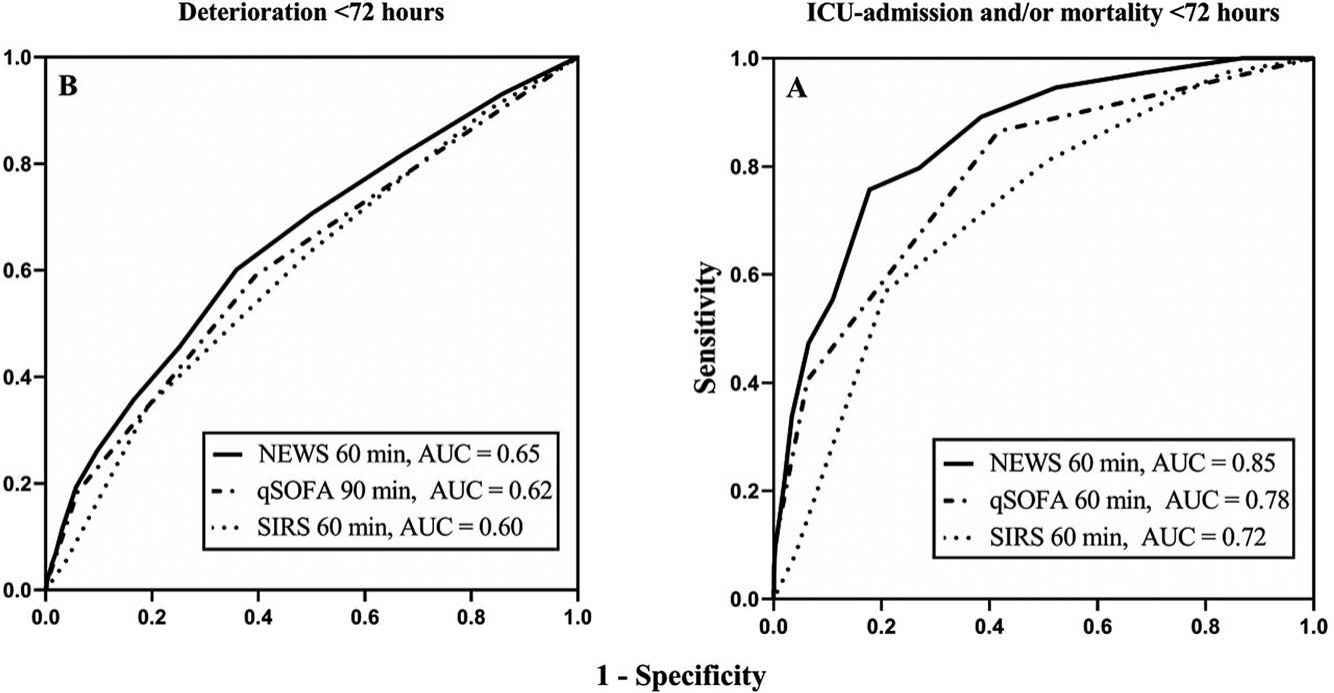
Receiver operating curves of the prediction of deterioration and mortality and/or ICU admission. The best performing measuring moment per score is showed in the figure. For deterioration (a), NEWS at 1 h (AUC, 0.65; 95% CI, 0.62–0.69), qSOFA at 90 min (AUC, 0.62; 95% CI, 0.59–0.65) and SIRS at 1 h (AUC, 0.60; 95% CI, 0.56–0.63). For ICU-admission or mortality (b), NEWS at 1 h (AUC, 0.85; 95% CI, 0.81–0.89), qSOFA at 1 h (AUC, 0.78; 95% CI, 0.73–0.84) and SIRS at 1 h (AUC, 0.72; 95% CI, 0.66–0.78). AUC, area under the ROC; CI, confidence interval; NEWS, national early warning score; qSOFA, quick sequential organ failure assessment; SIRS, systemic inflammatory response syndrome.

All three scores were predictive of deterioration, ICU-admission/mortality and a rise in SOFA score of at least two points (*P* < 0.05) (Fig. 2; Supplementary Tables 1–3, Supplemental digital content 1, http://links.lww.com/EJEM/A330). The NEWS had a higher prognostic accuracy to predict deterioration (i.e. development of organ failure, ICU-admission and/or mortality; *P* < 0.05) and ICU-admission/mortality (*P* < 0.001) compared with qSOFA and SIRS, whereas the prognostic accuracy to predict a rise in SOFA score of at least 2 points was not different between the three scores (Supplementary Table 4, Supplemental digital content 1, http://links.lww.com/EJEM/A330).

**Fig. 3:**
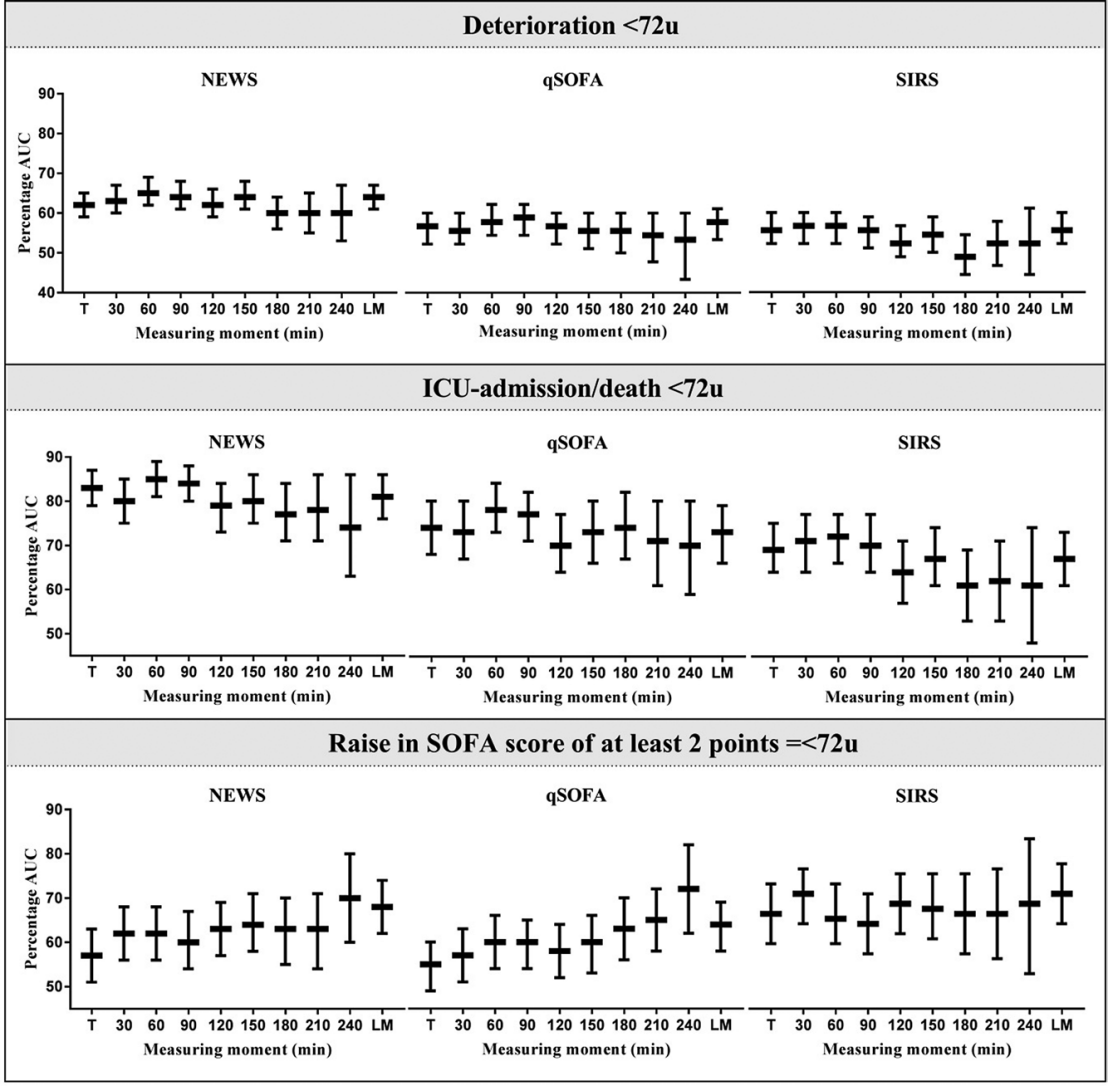
AUC for each individual score and measuring moment per different outcome. AUC with 95% confidence interval of the NEWS, qSOFA and SIRS per measuring moment, for all different outcomes. First row is the AUC for the prediction of deterioration <72 h, the second shows the AUC for the prediction of ICU-admission/mortality <72 h and the third shows the AUC for the prediction of a raise in SOFA score of at least 2 points <72 h. For exact number, see Supplemental Table 1–3. AUC, area under the ROC; NEWS, national early warning score; qSOFA, quick sequential organ failure assessment; SIRS, systemic inflammatory response syndrome.

NEWS reached the highest accuracy to predict deterioration [AUC, 0.65; 95% confidence interval (CI), 0.62–0.69; significant vs. triage] and ICU-admission/mortality at 1 h (AUC, 0.85; 95% CI, 0.81–0.89; NS different vs. triage) and to predict a rise in SOFA of at least 2 points at 4 h (AUC, 0.70; 95% CI, 0.60–0.80; significant vs. triage) (Figs 2 and 3; Supplementary Tables 1–3 and 5–7, Supplemental digital content 1, http://links.lww.com/EJEM/A330). Thus, the accuracy of NEWS to predict deterioration or a rise in SOFA score of at least 2 is higher at 1 h after triage compared with triage, whereas the association with ICU-admission/mortality is not different between time-points from triage up to ED discharge.

The prognostic accuracy of the qSOFA to predict deterioration or ICU-admission/mortality was highest at 90 min to predict deterioration (AUC, 0.62; 95% CI, 0.58–0.65; NS different vs. triage), at 1 h to predict ICU-admission/mortality (AUC, 0.78; 95% CI, 0.73–0.84; NS different vs. triage) and at 4 h to predict a rise in SOFA score of at least 2 (AUC, 0.72; 95% CI, 0.62–0.82; significant vs. triage) (Figs 2 and 3; Supplementary Tables 1–7, Supplemental digital content 1, http://links.lww.com/EJEM/A330). However, the accuracy of the qSOFA to predict these outcomes was not significantly different between the time-points from triage until ED discharge.

The accuracy of the SIRS score was highest at 1 h after arrival to predict deterioration (AUC, 0.60; 95% CI, 0.56–0.63, NS different vs. triage) and ICU-admission/mortality at (AUC, 0.72; 95% CI, 0.66–0.77; NS different vs. triage), and at 30 min after triage to predict a rise in SOFA score of at least 2 (AUC, 0.63; 95% CI, 0.57–0.68, *P* < 0.001; NS different vs. triage) (Figs 2 and 3; Supplementary Tables 1–7, Supplemental digital content 1, http://links.lww.com/EJEM/A330). Of the measurements obtained after triage, we had to omit measurements taken at 3 h and later due to insufficient power based on a post hoc power analysis (Supplementary Table 8, Supplemental digital content 1, http://links.lww.com/EJEM/A330). Thus, the time-point between triage and ED discharge of the SIRS score does not affect its accuracy.

### Association between change in the scores over time and deterioration

**Fig. 4:**
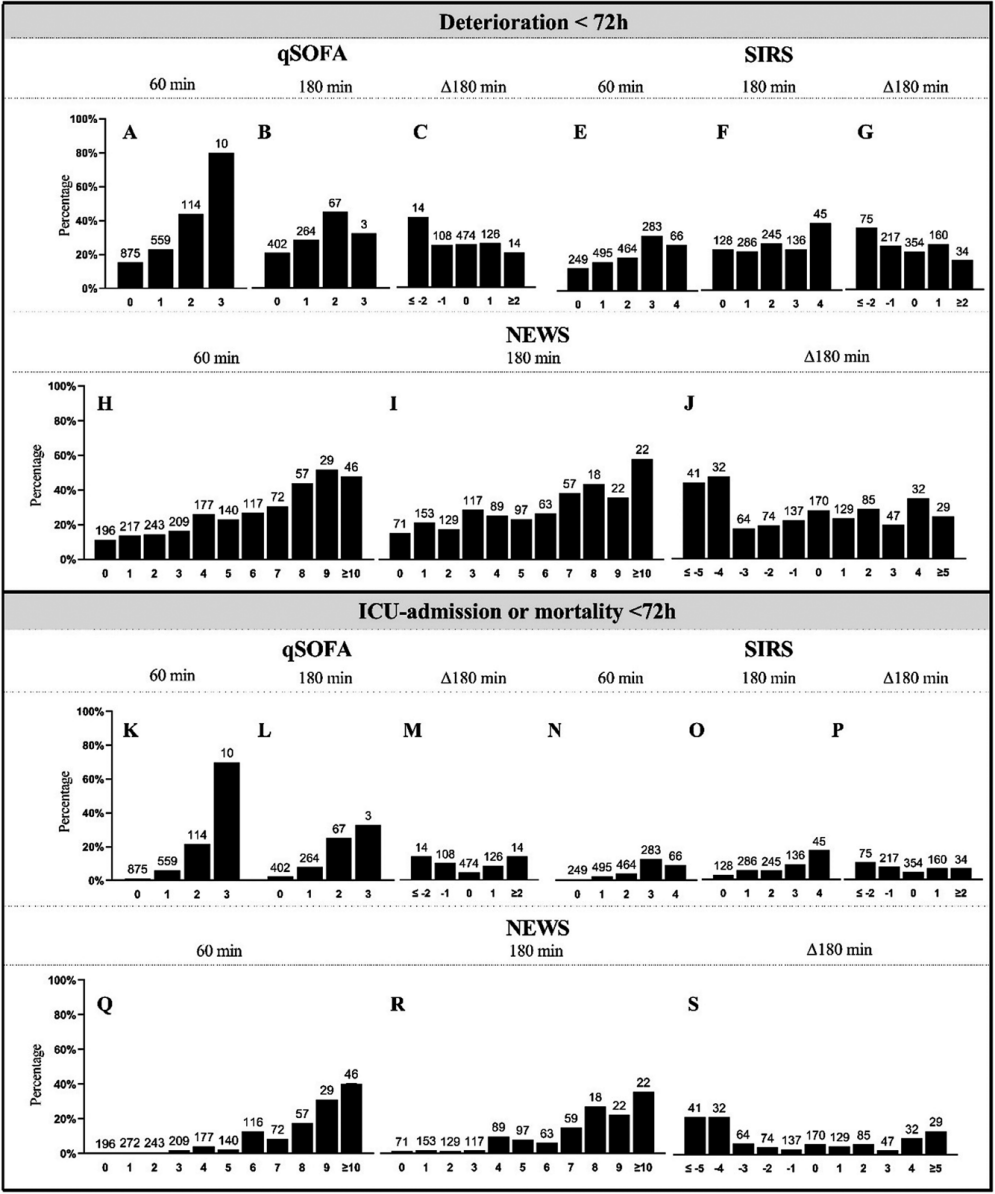
qSOFA, SIRS and NEWS at 1 h, 3 h and Δ3 h. Percentages per score per moment in time annotated for deterioration <72 h and ICU-admission/mortality <72 h. The sample size of each group is annotated above each bar. Δ180, 180 – triage; AUC, area under the ROC; NEWS, national early warning score; qSOFA, quick sequential organ failure assessment; SIRS, systemic inflammatory response syndrome.

To determine whether the change in NEWS, qSOFA or SIRS over time affects their prognostic accuracy for deterioration, the change in these scores from triage up to 3 h after arrival to the ED was analyzed. The NEWS score was unchanged in 170 patients (20.2%), lowered in 348 (41.4%) patients and rose in 322 (38.3%). The qSOFA was unchanged in 474 patients (64.4%), lowered in 122 (16.6%) patients and increased in 140 (19.0%). The SIRS score remained unchanged in 354 patients (42.1%) up to 3 h after ED arrival, decreased in 289 (25.8%) and rose in 194 (23.1%) patients (Fig. 4).

Patients whose scores abated were less likely to deteriorate. Among 73 patients with a decrease in NEWS of at least 4, 45.2% deteriorated and 21.9% went to the ICU or died, whereas among 61 patients with a rise in NEWS of at least 4, only 29.5% deteriorated and 11.5% went to the ICU or died. In line with these observations, 42.9% of patients with a decrease in qSOFA of at least 2 and 21.4% of patients with a rise in qSOFA of at least 2 deteriorated. Further, 37.3% of patients with a decrease in SIRS of at least 2 and 17.6% of patients with a similar rise in SIRS score deteriorated (Fig. 4). Next, we calculated the delta of each score from triage up to ED discharge at 30 min intervals and associated these with the outcomes. Only ΔNEWS up to ΔNEWS150, calculated by subtracting the NEWS at triage from the NEWS at the moment in time, was significantly associated with deterioration albeit with a very low predictive accuracy (Supplementary Table 9, Supplemental digital content 1, http://links.lww.com/EJEM/A330). Together, the NEWS and SIRS changed in the majority of the patients, in contrast to the qSOFA that remained unchanged in the majority; both a decrease as well as an increase in sepsis scores were associated with deterioration and ICU-admission/mortality.

## Discussion

In this post hoc analysis of prospectively collected data, the time-dependent effects on the prognostic accuracy of NEWS, qSOFA and SIRS to predict clinical deterioration and, as secondary outcomes, ICU-admission/mortality and a rise in SOFA score of at least 2 were assessed. Upon triage, all scores measured were associated with clinical deterioration, and the prognostic accuracy of the NEWS was higher compared with qSOFA and SIRS. The highest prognostic accuracy of the NEWS was reached at 1 h after triage compared with triage. As a clinical consequence, physicians working at the ED should best employ the NEWS at 1 h after ED arrival to predict the course in early sepsis and guide clinical decisions.

In this study, we demonstrated the accuracy of NEWS, qSOFA and SIRS to predict clinical deterioration defined as the composite endpoint of (multi)organ failure, ICU-admission or mortality, whereas previous studies mostly concentrated on mortality or a change in SOFA score. All scores performed moderate in predicting deterioration. As expected, based on the accuracy of the scores to predict mortality [2,12,18], SIRS had the highest sensitivity (63% sensitivity and 51% specificity), qSOFA had the highest specificity (18% sensitivity and 94% specificity), and NEWS had a more balanced accuracy (45% sensitivity and 75% specificity). Although being a secondary outcome in our study, the accuracy to predict mortality is in line with previous studies [13,14,18,19,20], where NEWS had a higher prognostic accuracy compared with qSOFA and SIRS to predict mortality (i.e. sensitivity 74% and specificity of 43%) [13,14]. Thus, consistent with previous studies describing the prognostic accuracy to predict mortality, we revealed qSOFA to have high specificity at the cost of low sensitivity, which may be explained by qSOFA lacking important variables (e.g. temperature and heart rate) [2,18]. NEWS outperforms both qSOFA and SIRS, probably due to the inclusion of mental status, blood pressure and oxygenation [18]. Based on their predictive accuracy, the qSOFA (high specificity) is suitable be used to identify patients at risk of deterioration, whereas SIRS (high sensitivity) can be used to identify patients not at risk of deterioration. Given the balance between sensitivity and specificity, combined with the AUC of the NEWS, we consider this the most suitable tool to facilitate early sepsis recognition at the ED.

Given the dynamic aspects of the clinical course in early sepsis as reflected by changes in vital parameters andconsequentlysepsis scores based on these parameters, timing of measurements will likely affect their prognostic accuracy. We hypothesized that a change in the sepsis scores during the ED stay would be more strongly associated with clinical deterioration as the score itself. This hypothesis was supported by the observation that a decrease in oxygen saturation or blood pressure among sepsis patients at the ED was associated with increased mortality [21,22]. Further, failure of normalization of vital signs among sepsis patients at the ED is associated with increased mortality [23]. We demonstrated ΔNEWS at 150 min and, hence, the change in NEWS from ED arrival up to 150 min, to significantly predict deterioration. These results are complementary to previous studies among sepsis patients at the ED: (a) a reduction in qSOFA among patients with qSOFA at least 2 [24] and (b) a reduction of NEWS among patients with NEWS at least 5 is associated with lower mortality risk [25]. It should be noted, however, that in the latter study, measurements were performed prehospital, during triage and at the ward and, hence, less frequently as in our current study.

Surprisingly, the highest risk of deterioration was found in groups with the largest change NEWS, qSOFA or SIRS, being either an increase or decrease, thereby annihilating the overall prognostic value of changes in risk scores. Not only patients with a large increase (i.e. ΔNEWS≥4, ΔqSOFA≥2 or ΔSIRS≥2) but also patients with a similar large decrease were at major risk for clinical deterioration and ICU-admission/mortality. Of note, in order to be able to have a large decrease, the initial score has to be high, and therefore, there is a high a priori chance of deterioration. Probably, the vital parameters that constitute the sepsis scores upon arrival to the ED reflect cellular injury that is not reversed by resuscitating at the ED, although this resuscitation might improve vital signs and decrease scores. Consequently, the initial improvement in vital parameters and sepsis scores may not be reflected by relevant improvement on a cellular level, leaving the patient at risk for further deterioration on a clinical level. ED staff should reassess the clinical condition and be aware that the risk for deterioration among patients with normalization of sepsis scores is dissimilar to those without abnormal scores.

Designing a strategy to stratify patients with early sepsis at the ED is essential for timely initiation of adequate care to prevent organ dysfunction and mortality. We demonstrate that almost one in four patients deteriorates within 72 h after admission. The prognostic accuracy of NEWS was higher than qSOFA and SIRS, and its accuracy increased over time, reaching its best predictive performance is 1 h after triage. Consequently, risk stratification of early sepsis patients should best be performed by reassessing the NEWS at multiple time-points.

## Limitations

The study population is limited to adult medical patients admitted for internal medicine (including nephrology, hematology, oncology, general medicine, allergology and infectiology), rheumatology, gastroenterology, pulmonology and emergency medicine. Patients admitted to the ED for other specializations were not screened as the incidence of infections and sepsis is very low among these patients. The primary outcome of the study is a composite outcome of organ failure, ICU-admission or mortality within 72 h after admission. Given that the majority of patients with sepsis are treated on the ward, either because ICU admission is not indicated yet based on the level of organ failure or ICU treatment is unwanted by the patient, we consider this composite outcome to capture deterioration in all patients. Since this outcome limits comparability with other studies, we have included more commonly used outcomes (i.e. ICU-admission and mortality <72 h) as secondary outcomes. To limit bias due to interobserver variability, all data used for this study are prospectively obtained by a trained team of medical student researchers, which may affect generalizability to routine clinical care, where values are obtained by different health care professionals. The scores were calculated post hoc using all available data for each time-point. Yet, we encountered missing data due to the fact that measurements were not obtained in individual patients or patients were already discharged from the ED. As such, the respiratory rate was frequently missing and had to be imputed in these cases. However, this reflects the reality of the ED and, therefore, makes the data relevant for real practice [26,27]. Further, this study was performed in an academic tertiary-care teaching hospital, which can limit generalizability to small rural hospitals. Nonetheless, this hospital has a substantial geographical spread in a rural area, ensuring a diverse population

### Conclusion

NEWS at triage has the best prognostic accuracy to predict clinical deterioration compared with qSOFA and SIRS. The prognostic accuracy of the NEWS was highest at 1 h after triage compared with triage. In addition to the association between the NEWS on one moment in time with deterioration, also the change in NEWS from triage up to 150 min was predictive of deterioration. Further, the incidence of clinical deterioration was even higher among patients with a decrease in sepsis scores compared with those with a rise in the score. Potentially, a reduction in the score might falsely reassure the physician, whereas a rise is considered as alarming.

## Acknowledgements

The authors wish to express their sincere acknowledgements to the UMCG sepsis team.

### Conflicts of interest

There are no conflicts of interest.

## Supplementary Material


