# Supplementary material for: Prognostic value of serial score measurements of the national early warning score, the quick sequential organ failure assessment and the systemic inflammatory response syndrome to predict clinical outcome in early sepsis
Source: Eur J Emerg Med. 2022 Jun 23;29(5):348–56. doi: 10.1097/MEJ.0000000000000924 (PMC9432814; doi:10.1097/MEJ.0000000000000924)
Supplement: Supplementary file 1 [file ejem-29-348-s001.pdf]

Supplemental Tables

Supplemental table 1. Discriminative performance of individual scoring systems per moment in time in predicting clinical deterioration <72h

**Clinical scoring system** (range)	**AUC (95% CI)**	**Cut-off point (≥)**	**Sens**	**Spec**	**PPV**	**NPV**
**NEWS** (0-20)						
**Triage**	0.62 (0.59-0.65)	5	43	75	30	83
**30 min**	0.63 (0.60-0.67)	5	48	71	30	84
**60 min**	**0.65 (0.62-0.69)**	5	45	75	32	84
**90 min**	0.64 (0.61-0.68)	5	48	75	34	84
**120 min**	0.62 (0.59-0.66)	5	44	73	32	82
**150 min**	0.64 (0.61-0.68)	5	36	81	37	80
**180 min3h**	0.60 (0.56-0.64)	5	43	70	33	78
**210 min**	0.60 (0.55-0.65)	5	44	66	33	76
**240 min**	0.60 (0.53-0.67)	5	47	66	37	74
**qSOFA** (0-3)						
**Triage**	0.60 (0.56-0.63)	2	14	95	42	81
**30 min**	0.59 (0.56-0.63)	2	19	93	40	81
**60 min**	0.61 (0.58-0.65)	2	18	95	47	82
**90 min**	**0.62 (0.58-0.65)**	2	18	94	45	81
**120 min**	0.60 (0.56-0.63)	2	16	94	45	79
**150 min**	0.59 (0.55-0.63)	2	18	93	46	78
**180 min**	0.59 (0.54-0.63)	2	16	93	46	76
**210 min**	0.58 (0.52-0.63)	2	15	90	37	73
**240 min**	0.57 (0.48-0.63)	2	17	90	45	69
**SIRS** (0-4)						
**Triage**	0.59 (0.56-0.63)	2	68	48	25	85
**30 min**	0.60 (0.56-0.63)	2	65	51	26	85
**60 min**	**0.60 (0.56-0.63)**	2	63	51	25	84
**90 min**	0.59 (0.55-0.62)	2	60	52	25	83
**120 min**	0.56 (0.53-0.60)	2	58	52	26	81
**150 min**	0.58 (0.54-0.62)	2	60	53	29	81

Deterioration; death, ICU-admission or development of organ failure (respiratory, liver and/or kidney) <72h. The range of the scoring systems are shown in the parentheses. The range of the scoring systems are shown in the parentheses. AUC: Area Under the Curve. ICU: Intensive Care Unit**.** CI: Confidence Interval. NEWS: National Early Warning Score. Sens: Sensitivity. SIRS: Systemic Inflammatory Response Syndrome. qSOFA: quick Sequential Organ Failure Assessment. Spec: Specificity. PPV: Positive Predictive Value. NPV: Negative Predictive Value. The associations between SIRS at 180 min and beyond with deterioration are omitted, due to lack of sufficient power (see Supplemental Table 8).

Supplemental table 2. Discriminative performance of individual scoring systems per moment in time in predicting mortality or ICU-admission <72h.

**Clinical scoring system** (range)	**AUC (95% CI)**	**Cut-off point (≥)**	**Sens**	**Spec**	**PPV**	**NPV**
**NEWS** (0-20)						
**Triage**	0.83 (0.79-0.87)	5	76	73	12	99
**30 min**	0.80 (0.75-0.85)	5	78	69	11	99
**60 min**	**0.85 (0.81-0.89)**	5	79	73	13	99
**90 min**	0.84 (0.80-0.88)	5	77	72	12	98
**120 min**	0.79 (0.73-0.84)	5	73	71	12	98
**150 min**	0.80 (0.75-0.86)	5	76	70	13	98
**180 min**	0.77 (0.71-0.84)	5	70	70	14	97
**210 min**	0.78 (0.71-0.86)	5	78	66	14	96
**240 min**	0.74 (0.63-0.86)	5	73	65	17	96
**qSOFA** (0-3)						
**Triage**	0.74 (0.68-0.80)	2	35	94	23	97
**30 min**	0.73 (0.67-0.80)	2	42	92	20	97
**60 min**	**0.78 (0.73-0.84)**	2	40	94	24	97
**90 min**	0.77 (0.71-0.82)	2	37	93	21	97
**120 min**	0.70 (0.64-0.77)	2	29	92	17	96
**150 min**	0.73 (0.66-0.80)	2	39	92	22	96
**180 min**	0.74 (0.67-0.82)	2	38	92	26	96
**210 min**	0.71 (0.61-0.80)	2	38	90	23	95
**240 min**	0.70 (0.59-0.80)	2	23	89	16	92
**SIRS** (0-4)						
**Triage**	0.69 (0.64-0.75)	2	85	46	7	99
**30 min**	0.71 (0.64-0.77)	2	82	49	7	98
**60 min**	**0.72 (0.66-0.77)**	2	81	49	7	98
**90 min**	0.70 (0.64-0.77)	2	76	51	7	98
**120 min**	0.64 (0.57-0.71)	2	67	51	7	97
**150 min**	0.67 (0.61-0.74)	2	74	51	8	97
**180 min**	0.61 (0.53-0.69)	2	63	50	8	95
**210 min**	0.62 (0.53-0.71)	2	65	47	8	95
**240 min**	0.61 (0.48-0.74)	2	65	49	11	94

AUC: Area Under the Curve. ICU: Intensive Care Unit**.** CI: Confidence Interval. NEWS: National Early Warning Score. Sens: Sensitivity. SIRS: Systemic Inflammatory Response Syndrome. qSOFA: quick Sequential Organ Failure Assessment. Spec: Specificity. PPV: Positive Predictive Value. NPV: Negative Predictive Value.

Supplemental table 3. Discriminative performance of individual scoring systems per moment in time in predicting a raise in SOFA score of at least 2 points

**Clinical scoring system** (range)	**AUC (95% CI)**	**Cut-off point (≥)**	**Sens**	**Spec**	**PPV**	**NPV**
**NEWS** (0-20)						
**Triage**	0.57 (0.51-0.63)	5	39	72	7	96
**30 min**	0.62 (0.56-0.68)	5	50	68	8	96
**60 min**	0.62 (0.56-0.68)	5	44	29	8	96
**90 min**	0.60 (0.54-0.67)	5	46	71	9	96
**120 min**	0.63 (0.57-0.69)	5	48	70	9	96
**150 min**	0.64 (0.58-0.71)	5	55	69	11	96
**180 min**	0.63 (0.55-0.70)	5	45	67	9	95
**210 min**	0.63 (0.54-0.71)	5	55	64	11	95
**240 min**	**0.70 (0.60-0.80)**	**5**	**64**	**64**	12	96
**qSOFA** (0-3)						
**Triage**	0.55 (0.49-0.60)	2	9	93	7	96
**30 min**	0.57 (0.51-0.63	2	20	91	10	95
**60 min**	0.60 (0.54-0.66)	2	16	92	11	95
**90 min**	0.60 (0.54-0.65)	2	16	92	10	95
**120 min**	0.58 (0.52-0.64)	2	18	92	12	95
**150 min**	0.60 (0.53-0.66)	2	79	91	14	94
**180 min**	0.63 (0.56-0.70)	2	19	91	14	94
**210 min**	0.65 (0.58-0.72)	2	16	89	10	93
**240 min**	**0.72 (0.62-0.82)**	**2**	**32**	**89**	23	93
**SIRS** (0-4)						
**Triage**	0.59 (0.53-0.65)	2	71	55	7	97
**30 min**	**0.63 (0.57-0.68)**	**2**	**77**	**51**	8	98
**60 min**	0.58 (0.53-0.65)	2	68	51	7	97
**90 min**	0.57 (0.51-0.63)	2	61	50	7	96
**120 min**	0.61 (0.55-0.67)	2	63	51	8	96
**150 min**	0.60 (0.54-0.67)	2	66	50	9	95
**180 min**	0.59 (0.51-0.67)	2	64	50	9	95
**210 min**	0.59 (0.50-0.68)	2	64	53	9	94
**240 min**	0.61 (0.47-0.74)	2	68	51	10	95

AUC: Area Under the Curve. CI: Confidence Interval. NEWS: National Early Warning Score. Sens: Sensitivity. SIRS: Systemic Inflammatory Response Syndrome. qSOFA: quick Sequential Organ Failure Assessment. Spec: Specificity. PPV: Positive Predictive Value. NPV: Negative Predictive Value.

Supplemental table 4. Paired-Sample Area Difference under the ROC Curve

Clinical scoring system	AUC difference	95% CI (≥) lower bound	95% CI (≥) upper bound	P-value
**Deterioration <72h**				
**NEWS -qSOFA**	0.023	0.000	0.045	0.045*
**NEWS-SIRS**	0.027	0.001	0.053	0.043*
**qSOFA-SIRS**	0.004	-0.029	0.036	0.816
**ICU-admission or death <72h**				
**NEWS -qSOFA**	0.092	0.051	0.133	<.001*
**NEWS-SIRS**	0.137	0.093	0.181	<.001*
**qSOFA-SIRS**	0.045	-0.019	0.109	.165
**Raise in SOFA score of at least 2 points <72h**				
**NEWS -qSOFA**	0.031	-.010	0.073	0.134
**NEWS-SIRS**	-0.019	-0.069	0.030	0.451
**qSOFA-SIRS**	-0.050	-0.110	0.009	0.098

Scores were compared using the measurements during triage per outcome. AUC: Area Under the Curve. ICU: Intensive Care Unit. CI: Confidence Interval. NEWS: National Early Warning Score. ROC: Receiver operating curve. SIRS: Systemic Inflammatory Response Syndrome. qSOFA: quick Sequential Organ Failure Assessment. *p<0.05.

Supplemental table 5. Comparison of the AUC of each score in the prediction of deterioration <72h

Clinical scoring system	AUC (95% CI)	AUC difference	95% CI of difference	P-value
**NEWS Triage vs**	0.62 (0.59-0.65)			
**30 min**	0.63 (0.60-0.67)	-0.018	-0.046-0.011	0.217
**60 min**	**0.65 (0.62-0.69)**	-0.034	-0.062-0.007	0.015*
**90 min**	0.64 (0.61-0.68)	-0.031	-0.062-0.000	0.054
**120 min**	0.62 (0.59-0.66)	-0.019	-0.051-0.014	0.256
**150 min**	0.64 (0.61-0.68)	-0.038	-0.070-0.005	0.024*
**180 min**	0.60 (0.56-0.64)	0.003	-0.036-0.042	0.892
**210 min**	0.60 (0.55-0.65)	-0.010	-0.060-0.039	0.678
**240 min**	0.60 (0.53-0.67)	-0.042	-0.114-0.030	0.249
**qSOFA Triage vs**	0.60 (0.56-0.63)			
**30 min**	0.59 (0.56-0.63)	0.005	-0.026-0.035	0.764
**60 min**	0.61 (0.58-0.65)	-0.015	-0.047-0.017	0.351
**90 min**	**0.62 (0.58-0.65)**	-0.022	-0.055-0.011	0.186
**120 min**	0.60 (0.56-0.63)	-0.004	-0.039-0.031	0.822
**150 min**	0.59 (0.55-0.63)	-0.005	-0.044-0.034	0.810
**180 min**	0.59 (0.54-0.63)	0.003	-0.039-0.044	0.904
**210 min**	0.58 (0.52-0.63)	0.022	-0.034-0.077	0.444
**240 min**	0.57 (0.48-0.63)	-0.014	-0.088-0.060	0.710
**SIRS Triage vs**	0.59 (0.56-0.63)			
**30 min**	0.60 (0.56-0.63)	-0.009	-0.037-0.018	0.505
**60 min**	**0.60 (0.56-0.63)**	-0.012	-0.040-0.016	0.394
**90 min**	0.59 (0.55-0.62)	-0.005	-0.035-0.025	0.744
**120 min**	0.56 (0.53-0.60)	0.019	-0.013-0.051	0.247
**150 min**	0.58 (0.54-0.62)	-0.010	-0.043-0.023	0.538

Comparison of the AUC of all three scores with their height during triage for the prediction of deterioration. AUC: Area Under the Curve. CI: Confidence Interval. NEWS: National Early Warning Score. SIRS: Systemic Inflammatory Response Syndrome. qSOFA: quick Sequential Organ Failure Assessment. The associations between SIRS at 180 min and beyond with deterioration are omitted, due to lack of sufficient power (see Supplemental Table 8). **p*<0.05.

Supplemental table 6. Comparison of the AUC of each score in the prediction of ICU-admission or death <72h

Clinical scoring system	AUC (95% CI)	AUC difference	95% CI of difference	P-value
**NEWS Triage vs**	0.83 (0.79-0.87)			
**30 min**	0.80 (0.75-0.85)	0.030	-0.016-0.075	0.201
**60 min**	**0.85 (0.81-0.89)**	-0.021	-0.062-0.020	0.316
**90 min**	0.84 (0.80-0.88)	-0.013	-0.050-0.024	0.209
**120 min**	0.79 (0.73-0.84)	0.023	-0.031-0.076	0.409
**150 min**	0.80 (0.75-0.86)	-0.009	-0.063-0.045	0.747
**180 min**	0.77 (0.71-0.84)	0.031	-0.031-0.092	0.328
**210 min**	0.78 (0.71-0.86)	0.017	-0.055-0.088	0.644
**240 min**	0.74 (0.63-0.86)	-0.016	-0.115-0.084	0.760
**qSOFA Triage vs**	0.74 (0.68-0.80)			
**30 min**	0.73 (0.67-0.80)	0.006	-0.053-0.065	0.837
**60 min**	**0.78 (0.73-0.84)**	-0.044	-0.104-0.015	0.143
**90 min**	0.77 (0.71-0.82)	-0.030	-0.092-0.033	0.352
**120 min**	0.70 (0.64-0.77)	0.018	-0.050-0.086	0.596
**150 min**	0.73 (0.66-0.80)	-0.019	-0.101-0.063	0.658
**180 min**	0.74 (0.67-0.82)	-0.012	-0.097-0.074	0.789
**210 min**	0.71 (0.61-0.80)	0.057	-0.046-0.159	0.278
**240 min**	0.70 (0.59-0.80)	-0.010	-0.140-0.121	0.886
**SIRS Triage vs**	0.69 (0.64-0.75)			
**30 min**	0.71 (0.64-0.77)	0.001	-0.053-0.054	0.984
**60 min**	**0.72 (0.66-0.77)**	-0.013	-0.065-0.038	0.611
**90 min**	0.70 (0.64-0.77)	-0.006	-0.063-0.051	0.842
**120 min**	0.64 (0.57-0.71)	0.038	-0.033-0.108	0.297
**150 min**	0.67 (0.61-0.74)	-0.016	-0.079-0.048	0.626
**180 min**	0.61 (0.53-0.69)	0.045	-0.026-0.116	0.212
**210 min**	0.62 (0.53-0.71)	0.032	-0.052-0.115	0.461
**240 min**	0.61 (0.48-0.74)	-0.028	-0.121-0.065	0.553

Comparison of the AUC of all three scores with their height during triage for the prediction of ICU-admission/mortality. AUC: Area Under the Curve. ICU: Intensive Care Unit**.** CI: Confidence Interval. NEWS: National Early Warning Score. SIRS: Systemic Inflammatory Response Syndrome. qSOFA: quick Sequential Organ Failure Assessment. *p<0.05.

Supplemental table 7. Comparison of the AUC of each score in the prediction of a raise in SOFA score of at least 2 points <72h

Clinical scoring system	AUC (95% CI)	AUC difference	95% CI of difference	P-value
**NEWS Triage vs**	0.57 (0.51-0.63)			
**30 min**	0.62 (0.56-0.68)	-0.048	-0.106-0.009	0.099
**60 min**	0.62 (0.56-0.68)	-0.046	-0.095-0.004	0.070
**90 min**	0.60 (0.54-0.67)	0.036	-0.089-0.017	0.187
**120 min**	0.63 (0.57-0.69)	-0.068	-0.128-0.008	0.026*
**150 min**	0.64 (0.58-0.71)	-0.074	-0.128- -0.019	0.008*
**180 min**	0.63 (0.55-0.70)	-0.061	-0.131-0.010	0.093
**210 min**	0.63 (0.54-0.71)	-0.038	-0.110-0.034	0.296
**240 min**	0.70 (0.60-0.80)	-0.137	-0.231-0.042	0.005*
**qSOFA Triage vs**	0.55 (0.49-0.60)			
**30 min**	0.57 (0.51-0.63	-0.022	-0.081-0.036	0.455
**60 min**	0.60 (0.54-0.66)	-0.055	-0.112-0.003	0.062
**90 min**	0.60 (0.54-0.65)	-0.054	-0.116-0.007	0.082
**120 min**	0.58 (0.52-0.64)	-0.045	-0.116-0.026	0.217
**150 min**	0.60 (0.53-0.66)	-0.064	-0.133-0.005	0.067
**180 min**	0.63 (0.56-0.70)	-0.115	-0.200-0.029	0.008
**210 min**	0.65 (0.58-0.72)	-0.114	-0.197-0.030	0.007*
**240 min**	0.72 (0.62-0.82)	-0.148	-0.260-0.035	0.010*
**SIRS Triage vs**	0.59 (0.53-0.65)			
**30 min**	0.63 (0.57-0.68)	-0.035	-0.096-0.026	0.265
**60 min**	0.58 (0.53-0.65)	0.005	-0.050-0.060	0.853
**90 min**	0.57 (0.51-0.63)	0.028	-0.026-0.082	0.308
**120 min**	0.61 (0.55-0.67)	-0.011	-0.075-0.054	0.748
**150 min**	0.60 (0.54-0.67)	-0.024	-0.092-0.044	0.487
**180 min**	0.59 (0.51-0.67)	0.006	-0.072-0.083	0.889
**210 min**	0.59 (0.50-0.68)	0.031	-0.052-0.113	0.468
**240 min**	0.61 (0.47-0.74)	0.018	-0.088-0.125	0.736

Comparison of the AUC of all three scores with their height during triage for the prediction of a raise in SOFA score of at least 2 points. AUC: Area Under the Curve. CI: Confidence Interval. NEWS: National Early Warning Score. SIRS: Systemic Inflammatory Response Syndrome. (q)SOFA: quick Sequential Organ Failure Assessment. *$p<0.05$.

Supplemental table 8. Post-hoc power analysis of scores at 180 min

Outcome	Score (180 min)	Sample size	p-value	Effect size	Observed Power
Deterioration	NEWS	840	0.000	0.028	0.999
	qSOFA	736	0.000	0.024	0.990
	SIRS	840	0.107	2.608	0.365*
ICU/mortality	NEWS	840	0.000	0.076	1.000
	qSOFA	736	0.000	0.066	1.000
	SIRS	840	0.001	0.012	0.892

Power analysis of each score measured at 180 minutes. All measurements had sufficient power, except for the SIRS in the prediction of deterioration. Intensive Care Unit**.** CI: Confidence Interval. NEWS: National Early Warning Score. SIRS: Systemic Inflammatory Response Syndrome. qSOFA: quick Sequential Organ Failure Assessment. *power was not sufficient

Supplemental table 9. Discriminative performance of Δ (moment in time – triage)

	Outcome			
	Deterioration <72h		ICU-admission or death <72h	
	**AUC (95% CI)**	**P-value**	**AUC (95% CI)**	**P-value**
**NEWS** (0-20)				
**Δ30 min**	0.51 (0.48-0.55)	.488	0.44 (0.36-0.51)	.065
**Δ60 min**	0.55 (0.51-0.57)	.043*	0.50 (0.45-0.58)	.943
**Δ90 min**	0.54 (0.51-0.56)	.049*	0.47 (0.39-0.55)	.359
**Δ120 min**	0.51 (0.47-0.55)	.541	0.47 (0.39-0.55)	.453
**Δ150 min**	0.54 (0.51-0.60)	.034*	0.49 (0.42-0.57)	.872
**Δ180 min**	0.49 (0.44-0.53)	.570	0.43 (0.34-0.52)	.075
**Δ210 min**	0.51 (0.46-0.57)	.629	0.46 (0.36-0.57)	.427
**Δ240 min**	0.55 (0.47-0.62)	.194	0.51 (0.39-0.63)	.831
**qSOFA** (0-3)				
**Δ30 min**	0.50 (0.46-0.53)	.867	0.49 (0.41-0.57)	.761
**Δ60 min**	0.52 (0.48-0.56)	.256	0.55 (0.48-0.63)	.123
**Δ90 min**	0.52 (0.48-0.55)	.323	0.50 (0.43-0.58)	.965
**Δ120 min**	0.50 (0.46-0.54)	.924	0.45 (0.37-0.54)	.196
**Δ150 min**	0.50 (0.46-0.54)	.958	0.49 (0.41-0.58)	.839
**Δ180 min**	0.49 (0.45-0.54)	.785	0.49 (0.39-0.59)	.839
**Δ210 min**	0.48 (0.42-0.53)	.367	0.42 (0.31-0.53)	.118
**Δ240 min**	0.51 (0.43-0.58)	.857	0.49 (0.36-0.62)	.868
**SIRS** (0-4)				
**Δ30 min**	0.50 (0.47-0.54)	.918	0.49 (0.42-0.56)	.796
**Δ60 min**	0.51 (0.47-0.54)	.797	0.50 (0.44-0.57)	.903
**Δ90 min**	0.50 (0.46-0.53)	.893	0.50 (0.43-0.57)	.888
**Δ120 min**	0.48 (0.44-0.51)	.210	0.48 (0.40-0.56)	.560
**Δ150 min**	0.51 (0.47-0.55)	.564	0.53 (0.45-0.60)	.474

Area under the Receiver Operating Curve of the difference in score per score and per moment in time. AUC: Area Under the Curve. ICU: Intensive Care Unit. CI: Confidence Interval. NEWS: National Early Warning Score. SIRS: Systemic Inflammatory Response Syndrome. qSOFA: quick Sequential Organ Failure Assessment. The associations between SIRS at 180 min and beyond with deterioration are omitted, due to lack of sufficient power (see Supplemental Table 8).
